# 3D printing in critical care: a narrative review

**DOI:** 10.1186/s41205-020-00081-6

**Published:** 2020-09-30

**Authors:** Mina Boshra, Justin Godbout, Jeffrey J. Perry, Andy Pan

**Affiliations:** 1 Faculty of Medicine, University of Ottawa, 451 Smyth Rd., Ottawa, ON K1H8M5 Canada; 2 Department of Emergency Medicine, Faculty of Medicine, University of Ottawa, 1053 Carling Avenue, Ottawa, ON K1Y 4E9 Canada; 3 Department of Emergency Medicine, The Ottawa Hospital Research Institute, 1053 Carling Avenue, Ottawa, Ontario K1Y 4E9 Canada; 4 Division of Critical Care Medicine, Department of Medicine, Montfort Hospital, 713 Montreal Road, Ottawa, ON K1K 0T2 Canada

**Keywords:** 3D printing, Critical care, Medical education, Clinical equipment, Patient care

## Abstract

**Background:**

3D printing (3DP) has gained interest in many fields of medicine including cardiology, plastic surgery, and urology due to its versatility, convenience, and low cost. However, critical care medicine, which is abundant with high acuity yet infrequent procedures, has not embraced 3DP as much as others. The discrepancy between the possible training or therapeutic uses of 3DP in critical care and what is currently utilized in other fields needs to be addressed.

**Objective:**

This narrative literature review describes the uses of 3DP in critical care that have been documented. It also discusses possible future directions based on recent technological advances.

**Methods:**

A literature search on PubMed was performed using keywords and Mesh terms for 3DP, critical care, and critical care skills.

**Results:**

Our search found that 3DP use in critical care fell under the major categories of medical education (23 papers), patient care (4 papers) and clinical equipment modification (4 papers). Medical education showed the use of 3DP in bronchoscopy, congenital heart disease, cricothyroidotomy, and medical imaging. On the other hand, patient care papers discussed 3DP use in wound care, personalized splints, and patient monitoring. Clinical equipment modification papers reported the use of 3DP to modify stethoscopes and laryngoscopes to improve their performance. Notably, we found that only 13 of the 31 papers were directly produced or studied by critical care physicians.

**Conclusion:**

The papers discussed provide examples of the possible utilities of 3DP in critical care. The relative scarcity of papers produced by critical care physicians may indicate barriers to 3DP implementation. However, technological advances such as point-of-care 3DP tools and the increased demand for 3DP during the recent COVID-19 pandemic may change 3DP implementation across the critical care field.

## Introduction

The advantages of the use of 3-Dimensionl printing (3DP) technology in the medical field are numerous [1, 2]. The capability of 3DP technology to create high fidelity products has proved to be an asset in the production of patient specific models and prostheses (e.g. congenital heart disease models based on a patient’s radiological data) [3]. Moreover, the digital design of 3D models can be easily altered to fit its intended use by utilizing widely available software [4–6]. Its high output speed and affordability of materials enables 3DP to meet high demands during shortages. For instance, it was able to supply many healthcare institutions with the protective equipment they needed during the COVID-19 pandemic [7]. Over the past few decades, many medical subspecialties began using 3DP for a variety of purposes. For example, cardiac surgeons began using computed tomography scans to create 3DP models of patients’ hearts to help with surgical planning [8]. This widespread use of 3DP in medicine has become prevalent enough to create special interest groups to devise appropriateness criteria of 3DP utilization in clinical settings. Through these criteria, 3DP implementation in medicine can become better regulated and therefore more established [3].

Nevertheless, despite the aforementioned uses and advantages, 3DP technology has not been as heavily implemented in the field of critical care. This is noteworthy since critical care has many areas where 3DP could be applied. One such area is simulation training. Simulation has been shown by multiple studies to be at least as efficient as standard lectures and visual aids [9, 10]. Likewise, it has been shown that the “See One, Do One, Teach One” approach to medical education should be replaced by a model emphasizing constant practice in order to achieve a high level of competency [11]. In the critical care field, procedural competence is often hindered by the virtue of its many risky yet infrequent procedures (e.g. cricothyroidotomy). These procedures, while relatively infrequent, carry higher risk for patients if done inappropriately. Therefore, simulation models would help increase the physician’s comfort with the procedure without causing any harm to patients. However, many commercially available simulators are either expensive or depend on animal substitutes (which present additional storage and procurement issues). Simulation using 3DP models can evade these issues due to their lower cost and ease of production [12]. Manufacturing of tools and equipment can also improve through 3DP implementation. This is especially important in low resource settings where acquiring medical equipment may be economically or logistically challenging. 3DP can also be used to educate patients, staff, or caregivers [13, 14].

We conducted a literature review to summarize the current education and therapeutic uses of 3DP for critical care procedures or within critical care settings.

## Methodology

The literature was collected by performing a comprehensive search of the PubMed database for all articles from inception until July 30th, 2019 containing the keywords used in literature to represent 3DP (e.g., 3D printing, three-dimensional print, additive manufacturing), and those used to represent critical care (e.g. critical care, emergency medicine, intensive care unit). The scarcity of the results led to the modification of the search strategy to include keywords representing the repertoire of skills of a critical care physician (Additional file 1). This modification was performed to include articles pertaining to the field of critical care but not necessarily by critical care physicians. With the assistance of a health sciences research librarian, terms and mesh categories for 3DP, critical care and skills required of a critical care physician were combined to create our search strategy (Additional file 1).

**Fig. 1:**
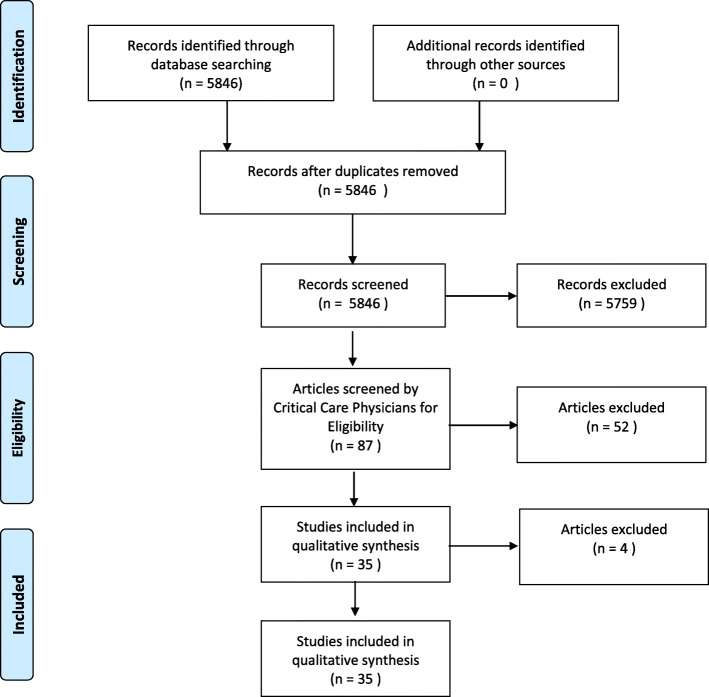
Prisma flow diagram of the number of papers found at each stage of the study

The search contributed 5846 results which were transported into Covidence (Veritas Health Innovation, Melbourne, Australia). The program found no duplicates. Papers were screened by title and abstract and disqualified if they had non-human subjects, implementations were not pertinent to critical care, or reviews. This created a list of 87 papers which were further reviewed for their applicability to the field of critical care by two critical care physicians (authors A.P. and J.G.). This left 35 papers which underwent full-text screening (Fig. 1). The papers were additionally examined for the degree of involvement of critical care in their production. Involvement in this study was defined as critical care physicians being part of the research team or research participants. Finally, the methodologies of the papers were divided into randomized control trials (RCT), technical reports, and quasi-experiments. Quasi-experiments were defined as studies that aim to demonstrate a causal relationship by introducing an intervention and control groups but without randomization [15].

## Results

Our search produced 31 papers that described possible uses of 3DP in critical care which can be divided into three main themes: Medical education (Med-Ed), patient care, and clinical equipment modification (CEM) (Table 1). Topics under Med-Ed included: bronchoscopy (9 studies), congenital heart disease (CHD) (4 studies), cricothyroidotomy (3 studies), and medical imaging (3 studies). Some single study utilities within medical education involved: thoracotomy, chest tube insertion, epistaxis management, and pediatric intubation. Studies within the patient care category included wound care (1 study), personalized splints (2 studies), and patient monitoring (1 study). CEM involved a 3DP stethoscope (1 study) and laryngoscope adjustments (3 studies). The characteristics of the papers can be found in Table 2.

**Table 1:** The uses of 3DP in critical care and their corresponding number of research papers

Major Topics	Utility	# of studies found
**Medical Education**	Bronchoscopy	9
	CHD	4
	Cricothyroidotomy	3
	Medical Imaging	3
	Thoracotomy	1
	Chest Tube Insertion	1
	Epistaxis Management	1
	Pediatric Intubation	1
	**Total**	**23**
**Patient Care**	Wound Care	1
	Personalized Splints	2
	Patient Lactose Monitor	1
	**Total**	**4**
**Clinical Equipment Modification**	Laryngoscope	3
	Stethoscope	1
	**Total**	**4**

**Table 2:** Study characteristics and major findings of 3DP utilities in critical care

First Author, et al.	Sub-category	Research design	Method	Population	sample size	Key Findings	Critical care Involved
Ho BHK et al. [16]	Bronchoscopy	Quasi experiment^a^	Qualitative evaluation of model by expert physicians using Likert questionnaire	resp physicians with over 6-years experience	5	The model was inexpensive, scored well with the experts, and provided an example of use of single stage printing of multi-material model which decreased production time. Next steps are to add vocal cords and changing the texture of the material to resemble human tissue.	No
Bustamante S. et al. [17]	Bronchoscopy	Technical Report	Comparison of bronchoscopy footage of model to real anatomy by authors	NA	NA	Similar to real human bronchoscopy, inexpensive, provides variety of both normal and anatomical variants (early take off of the right bronchus)	No
Al-Ramahi J. et al. [18]	Bronchoscopy	Technical report and Quasi experiment	Used formulas to create a model that replicated the hardness of tissue at different ages. Questionnaire and scale given to participants following trying the model	Otolaryngology staff and faculty	10	Produced a bronchoscopy model that was judged as accurate anatomically and simulated the change in tissue hardness as age changes. Provides an inexpensive, easy to replace model for training. Next step: adding humidification to the model to be more realistic.	No
Hornung A. et al. [19]	Bronchoscopy	Technical Report	Authors looking at footage using rigid and flexible bronchoscopy in water immersion	NA	NA	Using the model immersed in water created a realistic simulation of bronchoscopy in patients by decreasing the light reflection normally seen in plastic models.	No
Deboer E.M. et al. [20]	Bronchoscopy	Technical report and RCT	Model produced using silicone casing and color-coded lobes for feedback. RCT to determine improvement short and long term.	Pediatric residents and specialists	28 resident and 6 physicians	RCT showed improvement in confidence, accuracy, and speed of bronchoscopy compared to control. Validated by experts as realistic. 43 to 250% cheaper than commercial models.	No
Parotto M. at al [21].	Bronchoscopy	Technical report and quasi experiment	Created an inexpensive low-fidelity model. Used a pretest/post-test and feedback form.	Intensive care residents	NR	Increase in confidence, decrease in time taken per practice, increased post test score after using the model.	Yes
Ghazy A. et al. [22]	Bronchoscopy	Technical report and quasi experiment	Prototype for adult bronchoscopy was created. The time to complete task in sequential practices while removing in experienced vs novice users was measured.	cardiovascular residents	10	Simulator decreased time taken per each practice, increased proficiency for both groups, and was able to differentiate experienced from not. Both groups reached same level at the end.	No
Pedersen T.H. et al. [23]	Bronchoscopy	RCT	VAS score of realism, anatomical accuracy, and time to achieve tasks were measured as participants tried the 3DP model vs. commercial model.	18 anesthetists and 12 respirologists	30	3DP model group had significantly better scores than commercial models in realism and was non inferior in time taken to achieve tasks like aspiration. However, bronchial blocking was better in the commercial model.	No
Steinfort D.P. et al. [24]	Bronchoscopy	Quasi experiment	Conduct tests using validated mBSTAT tool of novice, intermediate and expert participants	28 med students, 3 resp. residents, 7 ICU residents, 6 resp. physicians	31 novice, 7 intermediate, 6 experts	3DP model was able to clearly differentiate scores between the various levels of experience. Next steps are to increase realism.	Yes
Costello J.P [25].	CHD	quasi experiment	pretest/post-test using Likert scale before and after seminar and simulation using a 3DP model	23 peds critical care residents	23	3DP model was shown to increase knowledge acquisition, reporting, and CHD structural conceptualization. Used malleable material to enable opening up the model and using stitches.	Yes
Olivieri L.J. et al. [26]	CHD	Quasi experiment	Likert questionnaire after session using 3DP models with open windows for inner anatomy.	22 residents, 10 ancillary workers, 38 nurses	70	3DP model increased confidence in management and understanding of anatomy especially in nurses. The increase was positively correlated to the difficulty of the case.	Yes
Olivieri L.J. et al. [27]	CHD	quasi experiment	Likert questionnaire after session using 3D virtual models and comparing the data to the previous study	19 physicians and 34 nurses	53	3DP model was shown to have higher scores in increased confidence in management and hand off but was more expensive than virtual models.	Yes
White S.C. et al. [28]	CHD	RCT	RCT comparing scores in a lecture only group vs lecture and model group for VSD and ToF	26 pediatric and otolaryngology/emerg residents in VSD, 34 in ToF	60	Study showed an increase in scores on a board-like exam after both intervention with higher scores for the 3DP group on ToF but higher for lecture only group on VSD.	Yes
Doucet G [29].	Cricothyroidotomy	Technical Report	Created a cricothyroidotomy model and used repeated expert feedback to modify it	NA	NA	Used multiple filaments to recreate the different density of tissues. Experts approved of its ability to teach students the major steps in the process	Yes
Hughes K.E [30].	Cricothyroidotomy	Technical report and quasi experiment	Created a cricothyroidotomy model that is perfused with fake blood, used 3 experts’ feedback, and did a pilot study using a convenience sample	52 EM docs	52	The model was reported to cause an improvement in confidence and the bleeding capability was rated as realistic. Next steps are to decrease the thickness of the outer membrane of the model and making the thyroid cartilage less obvious.	Yes
Katayama et al. [31]	Cricothyroidotomy	Technical report and RCT	Made a model using CT data. Used RCT to compare conventional model to 3DP model with a post-test using a porcine model.	52 residents	52	The results showed that the 3DP model was as efficacious as the conventional model but was much more inexpensive.	No
Risler Z. et al. [32]	Medical Imaging	Technical report	Made a model of dislocated shoulder in silicon casing and used US to test realism	NA	NA	The model produced realistic images and allows to see the dislocation as well as the needle during simulation of needle aspiration.	Yes
Javan R. et al. [33]	Medical Imaging	Technical Report	Made a model using various materials of different echogenicity and resonance to create a life like model of the neck for US and CT guided procedures	NA	NA	The model allows for training in thyroid and lymph node biopsies, internal jugular vein access, facet joint injections, and pain management interventions. Accurately presents different tissue densities. However, each part had to be fabricated separately.	No
O’Reilly M.K [34].	Medical Imaging	Technical report and quasi experiment	Created a high-fidelity model of femoral vessel with fake blood perfusion and a pulse. Used Likert scale questionnaire to judge it compared to other available models.	Interventional Radiology residents	19	The results showed that the model was realistic for both anatomical variant and diseased vessels. Scored higher than other models in realism. The feedback was used and they were able to modify the gelatin used to make it more stable and increase the compressibility of the venous system.	No
Yates E. et al. [35]	Thoracotomy	Technical report and quasi experiment	Created a thoracotomy model and tested it on EM residents using before and after questionnaires	EM residents	21	The questionnaires showed that the 3DP model increased their confidence of performing the procedure.	Yes
Bettega A.L. et al. [36]	Chest Tube Insertion	Technical report and RCT	Created a chest tube model and used medical students and pre/post questionnaires to assess.	Medical students	49	The study showed an increase in confidence equal to animal models.	Yes
Estomba C.C. et al. [37]	Epistaxis	Technical report and quasi experiment	Made a multi-material model with pulsating blood vessels and used a post questionnaire to assess it.	EM and ENT residents	NR	The model received positive feedback from the residents for its realism.	Yes
Park L. et al. [38]	Endotracheal intubation	Technical Report	Created a multi-material model to match the different tissue characteristics.	NA	NA	The model was feasible to make and inexpensive to produce. Nest steps for the model is to modify the trachea to increase its realism.	No
Muwaffak Z. et al. [39]	Wound Care	Technical report	Used a mixture of polycaprolactone and copper, zinc or silver to create personalized dressings. Measured the dose released across multiple days and its effects on *S. aureus* cultures.	NA	NA	The model released its content for 72 h, was adaptable to any shape on the body, was successful in limiting S. aureus growth.	No
Li J. et al. [40]	Splints	Technical report and quasi experiment	Used 3D scanner and an algorithm to create a personalized design of a splint with enough immobilization.	healthy volunteers	5	Successfully created personalized splints with minimal discomfort, good immobilization, fast rendition, made in 2–5 h. Next steps are to add Velcro straps to increase its flexibility to edema.	No
Wu P-K. et al. [41]	Splints	Case study	3D scanner used on a patient and printed splint for left arm immobilization after shoulder debridement.	patient	1	Using the splint was linked to increased healing speed, decreased swelling, and a faster recovery from surgery and infection. It was also of a similar price as traditional splint. However, it took 66 h to make.	No
Gómez-Ramos J.J. et al. [42]	Patient monitor	Technical report and quasi experiment	3D printed box with chemical reagents and connected to a smartphone. Compared to gold standard and on a volunteer undergoing physical activity.	Volunteer	1	Its measures were comparable to conventional lactate measure, showing that it can work in real time.	No
Aguilera-Astudillo C. et al. [43]	Stethoscope	technical report and quasi experiment	3D printed chest piece, a microphone, dongle and smartphone. Tried it on 4 patients.	Patients	4	Was able to pick up S1 and S2 on all four patients and record the sounds. Works great for rural areas and crowded emergency rooms. Next steps are to create an algorithm that processes the audio signals.	No
Kim S-H. et al. [44]	Laryngoscope	Technical report and RCT	Created a 3DP ergonomic handle. Used an RCT measuring success rate, intubation time, and subjective ease score while using the 3DP handle or conventional handles.	med students	40	The 3DP handle group had improved intubation time, ease score, and decreased number of trials needed on difficult scenarios compared to conventional handle.	Yes
Dinsmore M. et al. [45]	Laryngoscope	Technical Report	3D printed a laryngoscope handle with 4 tiles producing light using the handler’s body heat. Measured the illumination produced and its decay speed.	NA	NA	The 3DP handle was able to produce 200 Lux for more than 2 min which allows for easy intubation on normal airways. Next steps are to improve the amount of light produced and its duration.	No
Lee D.W. et al. [46]	Laryngoscope	Technical report and quasi experiment and RCT	3D printed attachment for smartphone and AirTraq to allow airway inspection without looking into the AirTraq. RCT performed on students to determine effect on intubation time and success rate. Quasi experiment on patients to determine safety and ability to broadcast the procedure.	Patients and Volunteers	40 volunteers and 30 patients	The results showed similar time to intubate and success rate between the model and the control. It was also used safely on all 30 patients. However, there was reported difficulty due to the need to manually zoom and the limitations caused by variability in bandwidth.	No

^a^Quasi-experiments were identified as studies introducing an intervention but without randomizing of the participants

Med-Ed papers accounted for 74% of the studies found. Bronchoscopy was the most common topic with 39% of Med-Ed papers, followed by CHD at 17.4%. Med-Ed papers were mostly technical reports and quasi-experiments with only 21.7% of the studies containing RCTs. On the other hand, 65.2% and 52.2% of the Med-Ed papers included a technical report or quasi experiment, respectively.

Patient care papers comprised 17.4% of the total number of papers with half focusing on personalized splints. Moreover, patient care was the only topic that included a case study while the other three papers were either technical reports or quasi-experiments. CEM papers also made 17.4% of the total number of papers found; however, CEM was mostly comprised of laryngoscope modification projects (75%). All CEM papers included a technical report with only one using an RCT.

**Fig. 2:**
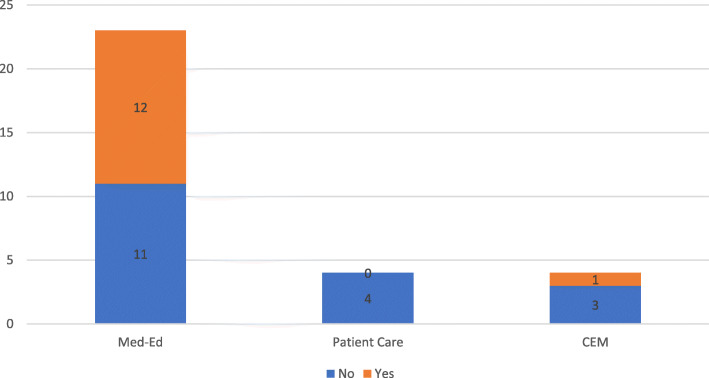
Number of studies involving critical care within the major utilities of 3DP in critical care

Comparing the involvement of critical care medicine in the papers, we found that only 13 of the papers had critical care contribution. Moreover, 12 papers were within Med-Ed while patient care and CEM having 0 and 1, respectively (Fig. 2).

## Discussion

### Results interpretation

This review shows that 3DP can have a variety of utilities in the field of critical care including medical education, patient care, and development of clinical equipment; however, Med-Ed takes the lead as the most common utility of 3DP with over 70% of the papers found discussing the use of 3DP models to train medical students and/or residents. This high percentage can be explained by the key findings of the papers. First, 3DP’s ability to create simulation models for numerous parts of the body including airways, shoulder girdle, and nasal cavity provides the opportunity to practice a large variety of skills. Such skills may be difficult to practice on real-life patients due to their high acuity and infrequency (e.g. cricothyroidotomy) [16–22, 32, 38]. Therefore, obtaining a model that can be used for frequent practice can be essential and life conserving [12]. Many of the studies showed that 3DP models were anatomically accurate and matched if not surpassed conventional models in their realism and student preference [18–20, 23, 28–32]. Moreover, the simulators were able to assess the difference in the proficiency between novices and experts by showing a clear correlation between the scores of the users and the number of years of experience [24]. This ability to discern between novices and experts enables the 3DP models to be used for assessing the competency of students as they progress in their training. In addition, the ability to help novice practitioners match experts after practicing on the simulator allows 3DP models to be useful training modules [22]. Another advantage of 3DP models is their ability to educate the user on both normal and variant/abnormal anatomy. For instance, 3DP models of congenital heart defects have been successfully used to increase the knowledge of participants of the anatomical issues and their consequences [25–28]. Normal anatomical variants have also been incorporated in many simulation models [17, 34].

Interestingly, the study by White and colleagues found that the 3DP group scored higher on the Tetralogy of Fallot test while the didactic class group scored better on the ventricular septal defect (VSD) test [28]. According to the authors, while VSD is simple enough to be learned through didactic learning, Tetralogy of Fallot is a complex case in which the tactile component may be advantageous to understanding the intricate anatomy. That is why the 3DP group were able to do better on that test than the classroom group. Therefore, for the optimum use of 3DP models in training, they should be used where a mix of visual and tactile information is beneficial.

Another noteworthy observation was that none of the papers we found discussed 3DP in critical care for adult cardiac disease. Although 3DP in cardiac medicine is a well-established field of research with various reviews [8, 47], 3DP utilization in adult cases mainly revolve around defect visualization, procedural planning, and surgical device innovation [47]. The scarcity of medical education utilities in adult cardiology is represented by both our results and those reported by Vukicevic and colleagues in their review of 3DP uses in cardiac medicine [47]. Considering the positive results of 3DP utilization in CHD for educating critical care physicians, similar training modules for the management of adult cases in cardiac intensive care units may prove beneficial.

The papers found under the categories of patient care and CEM represent the innovations possible through 3DP’s versatility. For example, the ability to create filaments of different characteristics allows for the production of more complex products. This was shown in the use of multiple materials to simulate the different tissue densities common to human structures [16, 33, 37, 38]. Furthermore, specially designed materials can be created for a particular utility. For instance, Muwaffak and colleagues were able to create specialized filaments containing silver, zinc, and copper and combine them into a personalized wound dressing that boasted the antimicrobial abilities of these metals [39]. Another source of versatility is the ability create 3DP molds of the desired structures. Using the sometimes-limited material properties offered by most 3DP technologies, 3DP molds can be used to shape silicon or gelatin to create anatomic structures that possess properties (e.g. mechanical) that more closely resemble tissue [20, 32]. 3DP molds were used with silicon in Risler and colleagues’ research to create the outer shoulder shell that provided feedback under ultrasound (US) that resembled human tissue [32]. 3DP versatility has also increased through personalization of therapeutic devices, such as splints to fit specifically to each patient. 3DP of personalized splints such as those described in Li et al. and Wu P-K et al. are made possible by hand-held 3D scanners that can capture the person’s exact measures within seconds, and which can then be used to custom-design the splint to fit the measured anatomy [40, 41].

Overall, 8 of the 31 studies specifically discussed that their 3DP models were either cheaper or of similar price to conventional models [16, 17, 31, 34, 35, 37, 38, 41]. This decrease in price can reach up to 250% which provides a strong motive for furthering the implementation of 3DP technology in critical care [17]. The expiration of the patent for various printers and the wide-spread availability of material has caused this decrease in cost and the increased availability of 3DP [48].

Despite the various advantages to 3DP implementation in critical care, only 13 of the 31 papers involved critical care physicians as authors or participants. This could be due to a variety of reasons from 3DP illiteracy, to lack of knowledge of possible implementations, to the fact that many 3D printed models are developed by surgeons or non-clinical researchers where the applications are more widespread. Another possible cause may be due to many physicians believing that the urgency of most critical care cases decreases the time available for designing and printing instruments. The relative difference in critical care papers found between the three topics supports this theory with 52% of Med-Ed papers involving critical care versus 25% and 0% in CEM and patient care respectively. This higher involvement in Med-Ed could be explained by the fact that education usually occurs in less urgent settings. However, with innovations such as 3DP wound dressings that can be made beforehand, printers that can produce splints in only a few hours, and the shortage of supplies mitigated by 3DP during the COVID-19 pandemic, we hope to see an increase in 3DP implementation in critical care tools and patient care [39, 40].

### Strengths and limitations

Our search strategy was expanded by searching for the use of 3DP in skills pertaining to critical care. This allowed us to capture and describe results from both current and possible implementations of 3DP in critical care. Moreover, we have presented the details and key findings of each study (Table 2) which can help guide future research. Many of the papers discussed were technical reports of models and hence can be developed and researched further. Additionally, our results were supported by the findings of other larger reviews [1, 49, 50].

Nonetheless, there are a few limitations to this review. First, our results are restricted to the papers found on the PubMed database. Moreover, since our search was conducted before the COVID-19 pandemic, additional uses of 3DP may have emerged to battle instrument shortage. Nevertheless, we believe that any extra papers would still fall under the major topics of Med-Ed, patient care, and CEM. Another limitation is the low number of papers found in both CEM and patient care. Furthermore, many of the papers found did not test the clinical significance of their innovations. However, the positive results from every quasi-experiment and RCT reported here supports the hypothesis that uses of 3DP are clinically significant. Further research guided by our description of the benefits of 3DP in critical care will also help mitigate some of the issues caused by the low number of results found.

### Future directions

With 3DP technology continuously improving, we expect a rise of new initiatives in the field of critical care. For instance, the ability of 3DP models to serve as simulation training modules for novice physicians will be crucial as the medical field begins its transition to competency-based learning. The versatility of 3DP raw materials makes it possible to create simulation models that cover an array of competencies and skills. For instance, researchers have been able to create high quality 3DP phantoms using different materials to resemble the physical characteristics of the distinctive tissue types [51]. These phantoms can be used to train novice critical care physicians on their imaging diagnostic skills as well as imaging-guided procedures [32, 33]. Nevertheless, further collaboration with 3DP companies is needed in the future to improve the fidelity of these phantoms through specially designed raw materials to more accurately depict the characteristics of human tissue [51]. With the continued development of 3DP simulation models, the authors hope that an open-source library with the printing files of the models can be made available so that physicians in resource-scarce regions can still maintain their training.

Furthermore, the tools used in critical care can benefit from the enhancements possible through 3DP. For example, biochemical research papers [52] have designed 3D printed materials that could be used to enhance wound healing. This ability can be applied to wound dressing manufacturing and tested in a critical care setting to determine the advantages they provide over commercially available dressings.

Another field in 3DP research that has been gaining attention is point-of-care testing (POCT). POCT is the field of diagnostic testing that can be done in real time generally outside of a laboratory and by untrained individuals [53]. This field has become essential for diagnosis both in the developing world and rural or resource scarce areas in the developed world [54]. Therefore, future research into 3DP POCT projects like ABO blood typing and wireless monitoring of key metabolites may be readily utilized in critical care settings [53, 55, 56].

The future implementation of 3DP in critical care has been affected greatly by the COVID-19 pandemic. The shortage of personal protective equipment and ventilation valves has supported the need for 3DP’s quick turnover and production rate [57]. Indeed, many research endeavours have utilized 3DP to overcome the scarcity of resources that faced many hospitals. For example, Callahan and colleagues were able to use 3DP to create nasal swabs that were comparable to the commercial ones [58]. Other uses of 3DP included production of face shields, N95 masks, ventilator valves, and environmental protection (ex. Hands-free door handles) [59, 60]. The pandemic was able to uncover the limitation of many of our hospitals when they were cut off their suppliers and faced shortage of necessary tools and equipments. However, this can be prevented in the future through two important steps. First, advocating for the development of 3DP labs within hospitals and the training of staff on the protocol for employment of 3DP tools during emergent situations may mitigate some of the effects of supply shortage. Moreover, the creation of a central depository for medical 3DP designs may help increase the access of hospitals to readily available products. Such a depository can also increase the number of trials a product undergoes which can hasten their development and improvement.

## Conclusion

This narrative review has summarized the major uses of 3DP in the field of critical care which were found to be mainly within the realms of medical education (e.g. simulation models and training modules), patient care (e.g. wound care and personalized splints), and clinical equipment modification (e.g. 3DP laryngoscope handle). Moreover, our search found that most of the research endeavours, while discussing 3DP utilities applicable to the field of critical care, were not performed by critical care medicine. This fact represents the need for critical care-specific studies that consider the needs of the field and how 3DP can fulfill them. Finally, we looked at how some of the new innovations in 3DP like biochemically active 3DP raw material may be beneficial for the future of critical care. With these various advantages of 3DP and the clear demand for its role in a plethora of aspects of critical care, we expect to witness a greater involvement of critical care physicians in this field in the near future.

## Supplementary information

**Additional file 1.**

## Data Availability

Not Applicable.

## Abbreviations

3DP: Three-dimensional printing
Med-Ed: Medical Education
MRI: Magnetic resonance imaging
CEM: Clinical Equipment Modification
CHD: Congenital heart disease
US: Ultrasound
RCT: Randomized Control Trial
VSD: Ventricular septum defect
