# Supplementary material for: 3D printing in critical care: a narrative review
Source: 3D Print Med. 2020 Sep 30;6:28. doi: 10.1186/s41205-020-00081-6 (PMC7525075; doi:10.1186/s41205-020-00081-6)
Supplement: Supplementary file 1 — Additional file 1. [file 41205_2020_81_MOESM1_ESM.docx]

Appendix A

Critical Care Skill List:

1) Bronchoscop

2) Intubation

3) Endotracheal intubation

4) Dislocation reduction

5) Shoulder dislocation

6) Laryngoscopy

7) Cricothyroidotomy

8) Flexible bronchoscopy

9) Airway stent

10) Wound dressing

11) Fracture

12) Suturing

13) Airway management

14) Primary care

15) Diagnosis

16) Injection

17) Wound Care

18) Chest tube

19) Tube thoracostomy

20) Thoracotomy

21) Intraosseous

22) Vascular access

23) Airway

24) Difficult airway/foreign body

25) central venous access

26) pigtail catheter

27) needle thoracostomy

28) thoracentesis

29) pericardiocentesis

30) paracentesis

31) pelvic binding

32) casting

33) splinting

34) tracheostomy

35) transvenous pacemaker

36) arterial line

37) nerve block

38) umbilical vein catheter

39) balloon tamponade

40) lumbar puncture

41) lateral canthotomy

42) resuscitative thoracotomy

43) perimortem cesarian section

44) dental splint

Appendix B

Search Strategy:

Search ((((((((((((((((((((((((((((((((((((((((((((((((((("critical care") OR "emergency care") OR "emergency medicine") OR "intensive care") OR intensive care unit*) OR emergency department*) OR emergency room*) OR CICU) OR point-of-care) OR ICU) OR bronchoscop*) OR intubation) OR "shoulder dislocation") OR laryngoscop*) OR cricothyroidotomy) OR stent) OR hemostat*) OR wound) OR fracture) OR suture) OR airway management) OR primary care) OR diagnosis) OR injection) OR "chest tube") OR intraosseous) OR thoracotomy) OR thoracostomy) OR vascular access) OR foreign body) OR "central venous access") OR "pigtail catheter") OR thoracentesis) OR pericardiocentesis) OR paracentesis) OR cast*) OR splint*) OR tracheostomy) OR pelvic binding) OR pacemaker) OR "arterial line") OR nerve block) OR "umbilical vein catheter") OR catheter) OR balloon tamponade) OR lumbar puncture) OR lateral

canthotomy) OR perimortem cesarian section) OR dental splint) OR ((((((("Emergency Medical Services"[Mesh] OR "Emergency Treatment"[Mesh]) OR "Critical Care"[Mesh]) OR "Emergency Service, Hospital"[Mesh]) OR ( "Intensive Care, Neonatal"[Mesh] OR "Intensive Care Units, Pediatric"[Mesh] OR "Intensive Care Units, Neonatal"[Mesh] OR "Intensive Care Units"[Mesh] )) OR "Bronchoscopy"[Majr]) OR ( "Wound Closure Techniques"[Mesh] OR "Wounds and Injuries"[Mesh] )) OR "Vascular Access Devices"[Majr]))) AND ((((((((((3D print*) OR 3D-print*) OR "additive manufacturing") OR Three-dimensional print*) OR "3D model") OR "3D simulation") OR Rapid prototype*) OR stereolithography) OR "3D display") OR ((("Printing, Three-Dimensional"[Majr]) OR "Printing, Three-Dimensional"[Mesh]) OR "Stereolithography"[Majr])) Sort by: Best Match
